# Advances in the study of CCT3 in malignant tumors: A review

**DOI:** 10.1097/MD.0000000000041069

**Published:** 2025-02-07

**Authors:** Yun-Feng Bai, Xiao-Hui Shi, Mo-Lemei Zhang, Jia-hui Gu, Ta-La Bai, Yin-Bao Bai

**Affiliations:** a Baotou Medical College, Baotou, Inner Mongolia, China; b Department of Thyroid Tumor Surgery, Inner Mongolia Autonomous Region People’s Hospital, Hohhot, Inner Mongolia, China.

**Keywords:** cancer, CCT, CCT3, research progress, tumor marker

## Abstract

Malignant tumors are among the leading causes of death worldwide, with their underlying mechanisms remaining largely unclear. Tumorigenesis is a complex process involving multiple factors, genes, and pathways. Tumor cells are characterized by abnormal proliferation, infiltration, invasion, and metastasis. Improving tumor diagnosis rates and identifying novel molecular therapeutic targets are of great significance for the advancement of modern medicine. Chaperonin containing TCP-1 subunit 3 (CCT3) is one of the subunits of the chaperonin containing TCP-1 complex, a molecular chaperone involved in protein folding and remodeling. CCT3 plays a crucial role in maintaining protein homeostasis, with key substrates including tubulin and actin. In recent years, CCT3 has been reported to be abnormally expressed in various cancers, correlating with prognosis and therapeutic outcomes. In this review, we summarize the basic structure and function of chaperonin containing TCP-1 complex and CCT3, and discuss the role of CCT3 in tumor development. Additionally, we explore its potential applications in cancer diagnosis and treatment.

## 1. Introduction

The protein homeostasis network refers to the balanced state achieved during the synthesis, folding, transport, aggregation, disaggregation, and degradation of proteins after their release from ribosomes. Molecular chaperones are critical mediators in protein homeostasis; they prevent adverse interactions that can arise during translation by shielding exposed hydrophobic amino acid residues. This prevents the formation of misfolded structures and enables the degradation or remodeling of incorrectly structured proteins, thus maintaining protein stability. Utilizing the energy derived from nucleotide binding and ATP hydrolysis, molecular chaperones enclose misfolded proteins within their central cavity, allowing them to fold correctly in isolation.^[1]^ Newly synthesized polypeptides typically engage in nonspecific interactions, and in the absence of chaperonin containing TCP-1 complex (CCT), they are prone to aggregation, leading to toxicity. Importantly, molecular chaperones are not part of the final functional structure of the proteins they assist. Molecular chaperones are products of a highly conserved gene family and are ubiquitous across various organisms. They play vital roles not only in protein folding but also in DNA replication, transcription, cytoskeletal dynamics, and signal transduction.^[2]^ The most significant of these is chaperonin, which exists as large complexes of approximately 1 MDa, primarily composed of 2 stacked oligomeric rings of 7 to 9 subunits, each featuring a central cavity structure. Each subunit consists of 3 domains: (1) the apical domain (A domain), responsible for substrate recognition and binding; (2) the equatorial domain (E domain), which binds ATP and forms inter-ring contacts; (3) the intermediate domain (I domain), which conveys conformational information between the first 2 domains.^[3]^ The intermediate and equatorial domains have highly conserved sequences, with variation primarily occurring in the apical domain. Molecular chaperonins are commonly classified into type I and type II. Type I chaperonins, found in bacteria, mitochondria, and chloroplasts, include heat shock proteins (HSPs) such as HSP110 (HSPH), HSP90 (HSPC), HSP70 (HSPA), HSP60 (HSPD1), HSP40 (DNAJ), and the small HSP family (HSP27). In *Escherichia coli*, GroES acts as a co-chaperone, binding to HSP10 in an ATP-dependent manner to form a “cap” structure that encapsulates the substrate and functions under stress conditions.^[4,5]^ Type II chaperonins are present in archaea and eukaryotes, such as the archaeal thermosome and CCT, and they utilize ATP to open and close their intrinsic “caps” to assist in protein folding. Notably, they can fold proteins in vitro without auxiliary proteins.

## 2. Structure and function of CCT and CCT3

CCT has the most complex structure among molecular chaperones,^[6]^ consisting of 2 stacked hetero-oligomeric rings formed by 16 subunit molecules in a “back-to-back” configuration. It is primarily composed of 8 different subunits: CCTα, CCTβ, CCTγ, CCTδ, CCTε, CCTζ, CCTη, and CCTθ (CCT1–CCT8). This arrangement forms a hollow cylindrical structure, characterized as a hetero-oligomeric protein, with each subunit having a molecular weight ranging from 50 to 60 kDa. The 8 subunits exhibit approximately 23% to 35% amino acid similarity, predominantly in their apical domains.^[7]^ It is well-known that the rapid proliferation of tumor cells necessitates a substantial synthesis of proteins. CCT may play a significant role in tumorigenesis by regulating cancer cell growth, apoptosis, and genomic instability.^[8]^ As a type II molecular chaperonin, CCT assists in protein folding, assembly, transport, and degradation through a complex network within cells. Additionally, CCT plays critical roles in DNA replication, transcription, intracellular signal transduction, and cytoskeletal functions. Beyond its essential function in proper folding to prevent protein misfolding, CCT also targets proteins for degradation. The folding of proteins by CCT requires the assistance of several co-factors and auxiliary folding proteins, including pre-folding proteins. Pre-folding protein is a heterohexameric chaperonin that works transiently with phosphoducin-like proteins to stabilize newly synthesized actin and tubulin proteins for transport into the central cavity of CCT.^[9]^ For proteins within cells to possess specific biological functions, they must adopt distinct three-dimensional structures. Misfolded or unfolded proteins not only lose their biological function but can also adversely affect normal cellular operations. CCT complexes are involved in folding approximately 10% of cytoplasmic peptides into functionally active proteins,^[10]^ with a wide variety of proteins participating in this process. The substrates folded by CCT can be categorized into several groups: essential substrates: these include actin and tubulin, which must rely on CCT for folding into their native conformations. Occasionally associated proteins: these proteins may require CCT activity intermittently, typically achieving their biological structure without CCT. Complex assembly proteins: some proteins utilize CCT as a platform for completing complex assemblies, such as E3 ubiquitin ligase (Von Hippel–Lindau [VHL]). Proteins regulating CCT activity: certain proteins can modulate the activity of CCT. CCT has been reported to be associated with numerous important mitotic proteins, including α-, β-, and γ-tubulins, α- and β-actins, cell division cycle protein 20 (CDC20), E-cadherin (CDH1), cyclin B, cyclin E, polo-like kinase 1, myosin, VHL, p53, KRAS, HRAS, p21ras, p27, signal transducer and activator of transcription 3 (STAT3), Notch, mammalian target of rapamycin (mTOR), Myelocytomatosis oncogene, luciferase, G-proteins, hepatitis B virus core protein, EBNA1 viral protein, eukaryotic translation initiation factor 3b, EIF3I, EIF3H, and members of the WD repeat protein family.^[11–16]^ Among these, actin and tubulin are major components of the cytoskeleton and are involved in cellular proliferation, cycle, and migration functions. Their activities are dependent on CCT. Additionally, some proteins associated with tumorigenesis, such as VHL, KRAS, p53, and STAT3, also require CCT for proper folding. Recent research has gradually uncovered the roles of various CCT subunits in tumor development. Currently, the chaperonin containing TCP-1 subunit 3 (CCT3) subunit has been linked to several malignancies, including breast cancer, lung cancer, gastric cancer, liver cancer, and cervical cancer.

CCT3, also known as CCTG, PIG48, TRIC5, CCT-gamma, or TCP-1-gamma (Table 1), plays an essential role in protein folding as one of the subunits of the CCT. The CCT3 gene is located on chromosome 1 (1q22),^[17]^ with a full-length cDNA of 2150 bp that consists of 16 exons, encoding a protein of 545 amino acids and approximately 60 kDa. CCT3 shares significant sequence similarity with other members of the CCT family and contains conserved structural domains with other distant chaperone proteins, playing a crucial role in protein homeostasis and proteomic stability. Tumorigenesis is a multifactorial process resulting from the dysregulation of gene expression in specific tissue cells, leading to abnormal cellular proliferation and the eventual production of tumorigenic proteins. Additionally, misfolding and improper assembly of proteins form the molecular basis of many diseases, including cancer.^[18]^ Each subunit of CCT, including CCT3, is vital for tumor formation, where the absence or overexpression of individual subunits can impact the overall biological function of the oligomeric complex. As a subunit of CCT, CCT3 is closely associated with the expression of tumor-related proteins, genes, and cell cycle regulatory proteins, such as cell cycle regulators and STAT3. In eukaryotes, CCT3 possesses 2 identical rings and may regulate microtubules and actin through various biological forms. Experiments involving the deletion of CCT3 have identified it as a novel regulator of spindle integrity, revealing that CCT3 deficiency induces defects in kinetochore–microtubule attachments, similar to the functions of other CCT subunits, Its loss leads to mitotic arrest.^[19]^ CCT facilitates the folding of cytoplasmic proteins, particularly key substrates like cytoskeletal proteins, actin, and tubulin. The correct folding of tubulin involves electrostatic and hydrophilic interactions between its N- and C-terminal domains and the A and I domains of the CCT3/6/8 subunits.^[3]^ The deficiency of tubulin due to CCT3 knockdown is one of the reasons for the inhibited cellular motility and induced cell cycle arrest,^[20]^ though further evidence is needed to support this hypothesis. In the context of human papillomavirus (HPV) infections, CCT3 and CCT2 knockdown resulted in reduced infection levels of 40% and 25%, respectively. Furthermore, it was found that HPV-16 L2 specifically interacts with endogenous CCT3 in mammalian cells, suggesting that HPV-16 L2 may bind exclusively to CCT3, which in turn complexes with other CCT subunits^[21]^; additional evidence is required to confirm this interaction. CCT3 primarily accumulates in the cytoplasm, with an increasing amount translocating to the nucleus. The reason for its nuclear translocation leading to different prognoses remains unclear. Research indicates that STAT3 is related to CCT3, with STAT3 participating in the biological activity of the eukaryotic chaperonin TRiC/CCT both in vitro and in vivo. The interaction between TRiC and STAT3 primarily occurs through the binding of CCT3 to the DNA-binding domain (DBD) of STAT3. Immunoprecipitation studies conducted in RRL show that only CCT3 consistently binds to and cross-links with STAT3. Moreover, upon IL-6 stimulation, STAT3 levels significantly decrease in the CCT3 knockdown group, suggesting that the binding of TRiC to STAT3 is partially mediated by CCT3, providing a protein-binding site during the folding of microtubules and STAT3.^[16]^ In tumors, it has been confirmed that silencing CCT3 can reduce both total STAT3 levels and phosphorylated STAT3 levels in HepG2 liver cancer cells.^[22]^ Additionally, CCT3 may influence tumor progression through the JAK/STAT3 pathway and is associated with drug resistance.^[23,24]^ Wang et al^[25]^ reported that anti-Karlinβ effectively inhibited CCT4-mediated STAT3 maturation, leading to disrupted STAT3 signaling and proteostasis. However, whether STAT3 specifically binds to a single subunit within the CCT requires further validation. Moreover, CCT3 has been shown to promote the nuclear translocation of β-catenin, likely recruiting it to the nucleus through direct binding.^[26]^ CCT3 plays a crucial role in maintaining cellular ATP levels and cytoplasmic translation. Reports indicate that glycolytic function is significantly impaired in CCT3-deficient cells, leading to a reduction of at least 25% in total ATP levels. Furthermore, the knockout of CCT3 also decreased protein translation, resulting in a significant reduction of the eukaryotic translation initiation factor 3 (EIF3G) protein. In EIF3G-deficient cells, protein synthesis and cell growth are compromised.^[20]^ CCT3 expression levels disrupt the energy supply balance within cells, affecting the intracellular levels of certain free amino acids involved in energy metabolism, such as: CCT3 expression levels impact glutamine levels, regulating apoptotic mechanisms. CCT3 may induce asparagine-mediated apoptosis. CCT3 regulates the membrane channels exchanging asparagine and other amino acids.^[27]^ Distinct subunits of CCT are believed to have unique substrate-binding specificities; for instance, CCT3 exhibits a higher affinity for Q/N-rich proteins compared to other subunits.^[28]^ These proteins can form cytoplasmic foci known as P-bodies, where untranslated mRNAs accumulate and are degraded. Changes in protein levels and structural model analyses suggest that the formation of P-bodies in mutated cells is associated with specific interactions between this subunit and Gln/Asn-rich fragments. In vitro gel shift assays indicate that mutations in CCT3 interfere with its ability to bind to Q/N-rich protein aggregates, thereby affecting CCT functionality. In studies conducted by Li et al,^[29]^ knockdown of CCT3 resulted in increased levels of CDK2 and CDK6 in gastric cancer. Conversely, another study indicated that the knockout of CCT8 significantly reduced CDK2 levels in liver cancer cells,^[30]^ although the reasons behind this substrate discrepancy remain unclear. Recent research has identified CCT3 as a novel regulatory factor for slc7a11, promoting tumor cell proliferation and growth through negative regulation of ferroptosis and positive regulation of the AKT signaling cascade.^[31]^ Furthermore, CCT3 is involved in various aspects of malignant tumor cell proliferation, cell cycle regulation, apoptosis, and migration, as well as assisting in the folding of cancer-associated proteins such as p53, VHL, and STAT3.

**Table 1:** The human CCT genes.

Name	Alternative names	Chromosome (location)	Exon count
CCT1	CCT1; CCTa; D6S230E; CCT-alpha; TCP-1-alpha	6 (6q25.3)	12
CCT2	CCTB; 99D8.1; PRO1633; CCT-beta; HEL-S-100n; TCP-1-beta	12 (12q15)	17
CCT3	CCTG; PIG48; TRIC5; CCT-gamma; TCP-1-gamma	1 (1q22)	16
CCT4	SRB; Cctd; CCT-DELTA	2 (2p15)	14
CCT5	CCTE; HEL-S-69; PNAS-102; CCT-epsilon; TCP-1-epsilon	5 (5p15.2)	13
CCT6A	CCT6; Cctz; HTR3; TCPZ; TCP20; MoDP-2; TTCP20; CCT-zeta; CCT-zeta-1; TCP-1-zeta	7 (7p11.2)	14
CCT6B	Cctz2; CCTZ-2; TSA303; CCT-zeta-2; TCP-1-zeta-2	17 (17q12)	14
CCT7	CCTH; CCTETA; NIP7-1; TCP1ETA	2 (2p13.2)	13
CCT8	Cctq; PRED71; D21S246; C21orf112	21 (21q21.3)	16

From the official NCBI gene database website (https://www.ncbi.nlm.nih.gov/).

CCT = TCP-1-containing chaperonin.

## 3. CCT3 and carcinogenicity-related factors

### 3.1. CCT3 and the cell cycle

The cell cycle is a critical step in cell proliferation, and its dysregulation is one of the hallmark features of uncontrolled tumor cell growth. The cell cycle consists of 4 phases: the G1 phase (pre-DNA synthesis), the S phase (DNA synthesis), the G2 phase (post-DNA synthesis), and the M phase (cell division). In mammals, cells primarily transition from G1 to S phase, then to G2 phase, before entering M phase for mitosis. Tumorigenesis is generally associated with the excessive activation or acceleration of the transition into the S phase.^[32]^ Proteins such as microtubule proteins, CDC20, and CDH1 act as substrates of CCT. Microtubule proteins increase during the transition from G1 to S phase and interact with CCT during the S phase. CDC20 and CDH1 play crucial roles from metaphase to anaphase.^[15,33]^ CCT3 collaborates with other CCT subunits to perform essential functions in cell division and cell cycle progression. Silencing CCT3 inhibits the proliferation and viability of tumor cells in breast cancer, lung cancer, liver cancer, melanoma, cervical cancer, and prostate cancer, leading to cell cycle arrest.^[19,20,27,34,35]^ Depletion of the CCT induces cell cycle arrest during mitosis and increases the percentage of abnormal mitotic cells. Deficiencies in 1 CCT subunit may disproportionately affect certain substrates compared to others. For instance, Li et al^[29]^ demonstrated that silencing CCT3 in gastric cancer increased the levels of CDK2 and CDK6. Other reports indicated that knocking down CCT8 significantly reduced CDK2 levels in hepatocellular carcinoma (HCC), suggesting substrate specificity variations among CCT subunits, the reasons for which remain unclear and warrant further investigation. Additionally, Zhang et al^[19]^ identified CCT3 as a novel regulator of spindle integrity, noting that the loss of CCT3 induces defects in kinetochore–microtubule attachments, paralleling the functions of other CCT subunits during mitosis. This deficiency can lead to mitotic arrest, which is crucial for proper kinetochore–microtubule attachment. Furthermore, silencing CCT3 increases the sensitivity of liver cancer cells to vincristine (a microtubule destabilizer) while decreasing their sensitivity to paclitaxel (a microtubule stabilizer). Given the role of CCT3 in the cell cycle and its influence on microtubule drug sensitivity, it is imperative to explore the upstream and downstream genes associated with it.

### 3.2. CCT3 and STAT3

**Figure 1.**
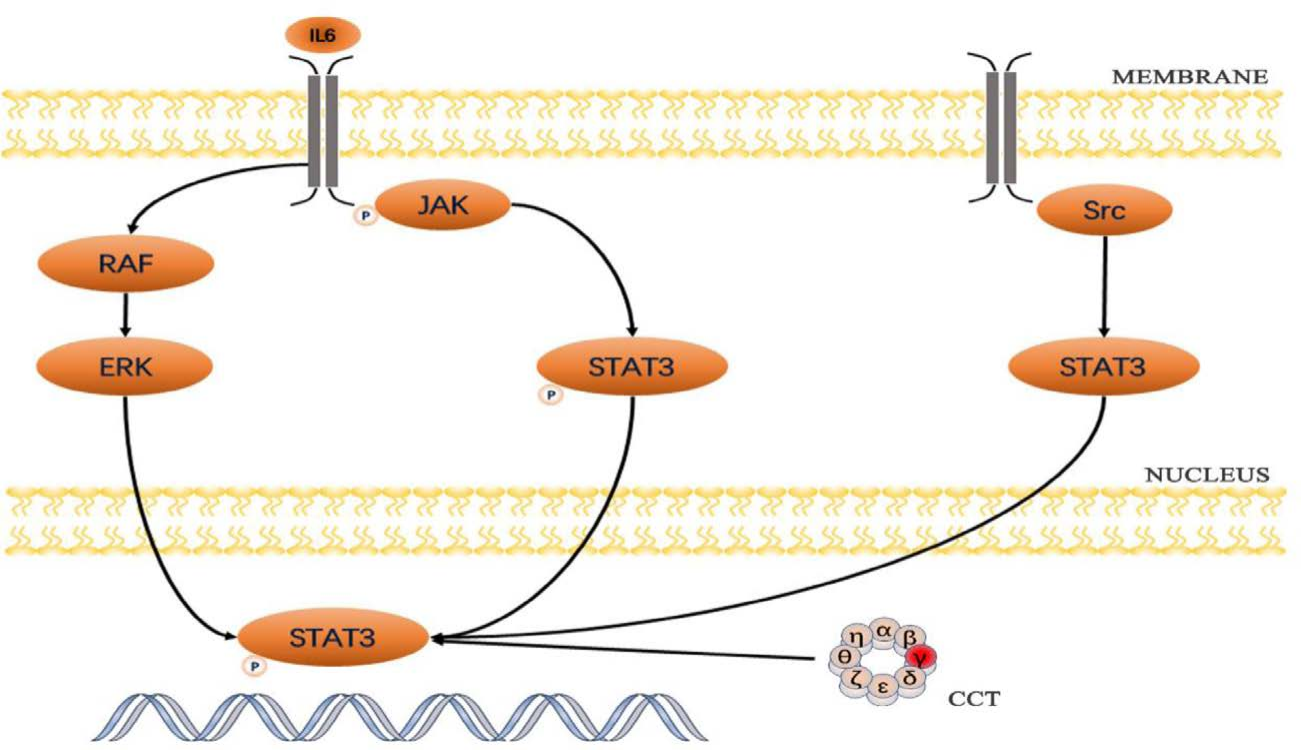
STAT3 signaling pathway. IL-6 is a cytokine that can activate STAT3 through the classical IL-6–JAK pathway; Src is a proto-oncogene, a non-receptor tyrosine kinase that can directly activate STAT3 independently of JAK; and abnormally activated Src relies on the activated STAT3 to play an oncogenic role. STAT3 = signal transducer and activator of transcription 3.

The signal transducers and activators of transcription (STAT) family includes STAT1, STAT2, STAT3, STAT4, STAT5a, STAT5b, and STAT6. The STAT family contains 6 functionally conserved structural domains: the N-terminal domain (NH2), coiled-coil domain (CCD), DBD, linker domain, Src homology 2 (SH2) domain, and C-terminal transactivation domain (TAD).^[36]^ Among these, STAT3 is an activation factor for various growth factors and signaling pathways, regulating physiological activities such as normal cell proliferation and apoptosis (Fig. 1). STAT3 is also involved in tumor proliferation, survival, and metastasis, and is closely related to drug resistance.^[24]^ Research by Kasembeli et al,^[16]^ has demonstrated that the structure and function of STAT3 in eukaryotic cells are regulated by CCT, which assists in the proper folding of unfolded or denatured STAT3 to acquire the functionality of native proteins. The primary subunit CCT3 interacts with the β-strand of the DBD of STAT3, with phosphorylation occurring at the 705 site under the mediation of interleukins, growth factors, and certain oncogenic proteins, activating STAT3 and leading to biological functions in downstream pathways. The DNA-DBD structure of STAT3 consists of 57% β-strands, forming a similar β-barrel structure to the DBDs of other members of the NF-κB/NF-at superfamily to which STAT3 belongs. Numerous studies have shown that STAT3 is in a persistently hyperactivated state in various tumors, particularly in advanced malignancies. Overactivated STAT3 can lead to the downregulation of immune-stimulating factors and is often found to interact with NF-κB, facilitating immune evasion by tumor cells.^[37]^ Cui et al^[22]^ experimentally verified that silencing CCT3 reduced the activity of STAT3 in the nucleus of liver cancer cells, even under IL-6 stimulation, indicating that silencing CCT3 negatively regulates the IL-6-STAT3 signaling pathway. This suggests that CCT3 may play a crucial role in the transport of STAT3 from the cytoplasm to the nucleus. Recent studies have indicated that CCT3 overexpression may influence tumor progression through the JAK–STAT3 pathway.^[23,34]^ Danni et al^[24]^ confirmed that silencing CCT3 can sensitize lung cancer cells to cisplatin by inhibiting the JAK2–STAT3 pathway, suggesting that CCT3 could be a novel target for overcoming cisplatin resistance in lung cancer patients. In the study by Wang et al,^[25]^ it was found that anti-Ca2+β effectively impeded CCT4-mediated STAT3 maturation, leading to disruptions in STAT3 signaling and protein homeostasis. While CCT3 can promote the folding and full activation of STAT3, whether this occurs through specific binding of an individual subunit within the CCT, thereby affecting the overall activity of CCT, requires further investigation.

### 3.3. CCT3 and P53

P53 is a well-known tumor suppressor and one of the most frequently mutated genes in cancer, located on chromosome 17p13.1. It consists of 11 exons and has a molecular weight of approximately 43,700 Da. As a tumor suppressor, P53 exerts its antitumor effects by regulating cell division, preventing the proliferation of cells with DNA mutations or damage, inducing ferroptosis, stabilizing the genome, and reducing tumor angiogenesis.^[38]^ Furthermore, P53 mutations are primarily missense mutations, accounting for about 70% of all p53 mutations. These mutations often result in changes to residues that specifically bind DNA, leading to single amino acid substitutions that impair the function of wild-type p53.^[39]^ In some cases, these mutations may confer new activities that allow tumor cells to bypass cell cycle checkpoints, enhancing their proliferation, division, invasion, and metastatic potential. Another mechanism by which p53 mutations inactivate the protein is by altering the conformation of the tetramer.^[40,41]^ Trinidad et al^[42]^ demonstrated that the folding of wild-type p53 is preserved through its interaction with CCT. A deficiency in CCT leads to the accumulation of misfolded p53, while mutated p53 can disrupt its interaction with CCT, resulting in conformational instability. Recent studies indicate that the N-terminal region of the p53 isoform Δ133p53 is critical for binding to CCT. CCT promotes the activation of Δ133p53β, and silencing CCT impairs the cell accumulation and migration induced by Δ133p53β.^[43]^ In colorectal cancer cells, the loss of p53 has been associated with the upregulation of phosphorylated CCT3.^[44]^ In liver cancer cells, inhibiting CCT3 results in DNA damage, as indicated by abnormal p53 levels in CRL-2329 cells.^[27]^ Recent research has shown that CCT3 mRNA expression is significantly higher in the p53 mutation group compared to the non-mutated p53 group in lung adenocarcinoma (LUAD).

### 3.4. CCT3 and NF-κB

The NF-κB family includes NF-κB1 (p105/p50), NF-κB2 (p100/p52), Rel A (p65), c-Rel, and Rel B.^[45]^ Among these, p65 is primarily localized in the cytoplasm and possesses a highly conserved Rel domain at its N-terminus, which is central to inflammation and immunity. It is highly expressed in various cancers and can transcriptionally activate downstream genes, playing a crucial role in regulating cell proliferation, apoptosis, and immune responses. In the cytoplasm, NF-κB dimers (p65 and p50) bind to IκB to form a trimeric complex, rendering NF-κB inactive. Upon stimulation by upstream signaling factors, IκB undergoes phosphorylation, leading to the rapid translocation of NF-κB into the nucleus, where it binds to DNA through the Rel domain, triggering a cascade of reactions.^[46]^ The NF-κB signaling pathway is closely associated with the expression and development of inflammatory factors and immune cells. In cancer, it is linked to tumor malignancy, tumor cell proliferation, apoptosis, angiogenesis, invasion, metastasis, and drug resistance.^[47]^ Recent studies by Ren et al,^[48]^ have shown that FBXW2 inhibits tumor cell stemness and paclitaxel resistance in breast cancer by promoting the ubiquitination of NF-κB-p65. Pejanovic et al^[49]^ confirmed that silencing CCT inhibits the transcription of IκB during the early stages of NF-κB signaling, reducing the acetyltransferase activity of p65 and potentially participating in the termination of NF-κB signaling. In an NF-κB-p65 rescue experiment, the overexpression of NF-κB-p65 was shown to rescue cell proliferation and migration in breast cancer cells affected by CCT3 knockdown.^[50]^ In cervical cancer cells, silencing CCT3 through bioinformatics analysis revealed that NF-κB-p65 is associated with tumor signaling pathways.^[35]^ Thus, further exploration of the relationship between CCT3 and the NF-κB signaling pathway may provide a viable research direction for understanding the mechanisms of tumor cell malignant transformation. Ren et al^[48]^ showed that FBXW2 affects breast cancer progression by inhibiting tumor cell stemness and paclitaxel resistance through ubiquitination of NF-κB p65. Pejanovic N et al^[49]^ demonstrated that silencing CCT inhibited IκB transcription at the early stage of NF-κB and reduced p65 acetyltransferase activity, which may thereby be involved in terminating NF-κB signaling. Overexpression of NF-κB-p65 rescued CCT3-affected cell proliferation and migration in breast cancer cells, as confirmed in an NF-κB-p65 rescue assay.^[50]^ Silencing of CCT3 in cervical cancer cells was detected by bioinformatics analysis, which found that NF-κB-p65 was associated with tumor signaling pathways.^[35]^ Therefore, further exploration of the relationship between CCT3 and the NF-κB signaling pathway may be a feasible research direction for the mechanism of malignant transformation of tumor cells.

### 3.5. CCT3 and Wnt/β-catenin

Wnt is a secretory glycoprotein characterized by a dual structural domain composed of the N-terminal domain and the C-terminal domain. Wnt signaling plays a crucial role in the initiation and progression of various types of cancer.^[51]^ Wnt signal transduction is classified into canonical and noncanonical pathways. The canonical pathway, also known as the β-catenin-dependent Wnt pathway, involves Wnt ligands (Wnt1, 2, 3, 3a, 8a, 8b, 10a, 10b) binding to receptors on the cell membrane, which leads to the phosphorylation of LRP. This results in an increase in β-catenin levels, allowing it to enter the nucleus and initiate a cascade of reactions that contribute to cell survival, proliferation, differentiation, and migration. The noncanonical pathway, or the β-catenin-independent Wnt pathway, involves Wnt ligands (Wnt4, 5a, 5b, 7a, 11) binding to cell surface receptors, activating downstream factors that regulate cytoskeletal reorganization and participate in cell proliferation, polarity, invasion, migration, and immune response.^[52,53]^ Research by Tang et al,^[54]^ has shown that p53 can bind to promoter site 2 of Wnt7b, and CCT influences Wnt7b expression through its interaction with p53. Silencing CCT significantly reduced the expression of Wnt7b and β-catenin while increasing the expression of cdh1, vimentin, c-myc, and cyclin D1. This indicates that knocking down CCT can inhibit the Wnt/β-catenin signaling pathway activated by Wnt7b, subsequently affecting the proliferation and migration of liver cancer cells. Furthermore, Qu et al^[26]^ confirmed that overexpression of CCT3 in breast cancer cells leads to increased levels of β-catenin, promoting its nuclear translocation. When CCT3 is knocked down, the Wnt/β-catenin signaling pathway, along with downstream target genes such as cyclin D1 and c-myc, is suppressed, ultimately affecting cancer cell proliferation and tumorigenesis. Additionally, a report indicated that knocking down circCCT3 (derived from exons 3–5 of CCT3 through back-splicing) inhibits the invasion of rectal cancer cells and induces apoptosis via the miR-613/WNT3 or VEGFA pathways.^[55]^ Moreover, studies have found that the expression of CCT3 in multiple myeloma (MM) is associated with the Wnt signaling pathway.^[23]^

### 3.6. CCT3 and AKT/mTOR

Protein kinase B (AKT), a serine–threonine protein kinase, consists of 3 isoforms: AKT1, AKT2, and AKT3. Upon phosphorylation, AKT activates downstream target proteins, such as mTOR.^[56]^ The mammalian target of rapamycin (mTOR) kinase forms 2 complexes, mTORC1 and mTORC2, composed of 2 subunits: mLST8 and Raptor. These subunits act as β-propeller proteins that stabilize mTOR kinase and recruit substrates. CCT facilitates the assembly and signaling of mTORC, with mLST8 binding to CCT deep within the folding chamber between 2 CCT rings, interacting primarily with the disordered N-terminal and C-terminal regions of specific CCT subunits.^[57]^ The PI3K–AKT–mTOR pathway plays a crucial role in regulating tumor cell growth, proliferation, survival, angiogenesis, invasion, and glucose metabolism.^[58]^ Numerous studies have shown that CCT regulates tumor cell proliferation, apoptosis, metastasis, autophagy, and chemoresistance through the PI3K/AKT/mTOR pathway.^[59–61]^ Chen X et al^[62]^ demonstrated that CCT expression levels are positively correlated with the phosphorylation of AKT and mTOR, inhibiting autophagy and drug-induced apoptosis via the regulation of the AKT/mTOR signaling cascade. Additionally, overexpression of CCT3 promotes the growth of LUAD cells by activating AKT. Inhibition of AKT in LUAD cells with either low or high CCT3 expression showed that cell proliferation was more significantly suppressed in CCT3-overexpressing cells.^[31]^ Bioinformatic analysis further revealed that the PI3K/AKT/mTOR signaling pathway is associated with CCT3 expression in head and neck squamous cell carcinoma cells.^[63]^ Taken together, CCT3 may influence tumor progression through the AKT/mTOR signaling pathway and represents a potential therapeutic target for cancer treatment.

## 4. CCT3 and cancer

### 4.1. CCT3 and liver cancer

Liver cancer is the fourth leading cause of cancer-related deaths worldwide, with a mortality rate closely mirroring its incidence. Due to the asymptomatic nature and early metastasis, most patients are diagnosed at an advanced stage, resulting in poor prognosis and often ineffective treatment.^[64]^ As early as 2002, Midorikawa Y et al^[65]^ detected high CCT3 expression in HCC with varying degrees of differentiation, closely correlating with tumor dedifferentiation. Wong N et al^[66]^ identified CCT3 in the 1q21-q22 region of HCC, a region associated with metastatic and more aggressive tumor phenotypes, with higher expression levels in tumor tissues compared to adjacent normal tissues. Subsequent mRNA expression analysis confirmed that CCT3 is localized at 1q22.^[17]^ Cui X et al^[22]^ demonstrated that CCT3 mRNA and protein levels are upregulated in HCC tissues compared to adjacent normal tissues, and silencing CCT3 inhibits cell proliferation, cell cycle invasion ability, and induces cell death. Additionally, silencing CCT3 resulted in downregulation of STAT3 and phosphorylated STAT3 (p-STAT3) expression, suggesting that CCT3 plays a key role in HCC progression by facilitating the transport of phosphorylated STAT3 from the cytoplasm to the nucleus, thus impacting the STAT3 signaling pathway. This finding provides new insights for targeting activated STAT3 in HCC treatment. Moreover, CCT3 has been identified as a novel spindle integrity regulator, similar to other CCT subunits, playing a crucial role in regulating microtubule structure and function (through kinetochore capture). Its high expression is associated with poor patient survival, and silencing CCT3 increases the sensitivity of HCC cells to microtubule-destabilizing drugs such as vincristine,^[19]^ providing a potential drug target for HCC therapy. In the same year, Qian EN et al^[67]^ demonstrated through ELISA analysis of peripheral blood samples that CCT3 has higher sensitivity in diagnosing HCC compared to alpha-fetoprotein, especially in alpha-fetoprotein-negative and early-stage HCC patients. CCT3 expression levels were correlated with etiology, tumor size, and pathological stage. Bioinformatics analysis revealed that CCT3 mRNA and protein expression levels are higher in HCC tissues than in non-HCC tissues, with strong discriminatory power between cancerous and noncancerous tissues. Its mRNA expression is negatively correlated with DNA methylation, and the most common mutation type is missense mutations, which affect HCC development through the cell cycle and DNA replication pathways.^[68]^ Liu Y et al^[69]^ found that CCT3 blocks PCBP2-induced ubiquitination of YAP and TFCP2 through the βTrCP E3 ligase pathway, extending the half-life of YAP and TFCP2. CCT3 is upstream of YAP and TFCP2, with positively correlated expression levels, indicating a role in HCC progression. The diagnostic capability of CCT3 is superior to alpha-fetoprotein to some extent, and targeting CCT3 may be an effective treatment for YAP-related liver cancer. In recent years, circ-CCT3, derived from the back-splicing of exons 3 to 5 of CCT3, has been found to promote HCC progression by regulating TEA domain transcription factor 1 (TEAD1) expression via sponging miR-1287-5p.^[70]^ Furthermore, Liu H et al^[71]^ showed that circ-CCT3 knockdown inhibits HCC growth, metastasis, invasion, and angiogenesis through the miR-378a-3p-FLT-1 pathway, and high circ-CCT3 expression is associated with poor prognosis and is an independent risk factor for overall survival in HCC patients. Additional studies suggest that CCT3-LINC00326, a type of RNA-binding protein, interferes with the CCT3-LINC00326 network, reducing lipid accumulation, increasing lipid degradation, and decreasing tumor growth both in vivo and in vitro.^[72]^ In summary, CCT3 is involved in HCC progression and serves as a potential biomarker and therapeutic target.

### 4.2. CCT3 and lung cancer

The incidence and mortality of lung cancer in China have been increasing yearly, with approximately 75% of patients dying within 5 years of diagnosis. The incidence is higher in women than men, but men are at greater risk. Additionally, the disease burden is greater in eastern China compared to the west. The risk factors for lung cancer include smoking, environmental and occupational exposure, chronic lung disease, pulmonary infections, and lifestyle factors. Early diagnosis, prognosis prediction, drug response assessment, and immunotherapy are crucial strategies for reducing mortality.^[73,74]^ As early as 2009, Keenan J et al^[75]^ found that CCT3 is associated with azithromycin resistance in lung squamous cell carcinoma (DLKP cell line). In recent years, data from the Cancer Genome Atlas and Genotype-Tissue Expression databases have shown that high CCT3 expression is associated with poor prognosis in LUAD patients.^[76]^ Based on these findings, many researchers have explored the role of CCT3 in lung cancer. Danni X et al^[24]^ analyzed the Gene Expression Profiling Interactive Analysis database and found that CCT3 is significantly upregulated in both LUAD and lung squamous cell carcinoma tissues. Moreover, LUAD patients with low CCT3 expression have significantly higher overall survival rates than those with high CCT3 expression. Silencing CCT3 inhibits LUAD cell proliferation, cell cycle progression, invasion, and migration, while also inducing apoptosis. Knockdown of CCT3 re-sensitizes LUAD cells to cisplatin by modulating the JAK2/STAT3 signaling pathway, suggesting that CCT3 may be a potential target for overcoming cisplatin resistance in LUAD patients. Shi H et al^[77]^ discovered that in addition to its impact on cellular phenotypes, silencing CCT3 also reduces YAP1 expression in non-small cell lung cancer, exhibiting antitumor effects. This was validated in in vitro xenograft mouse models, where silencing CCT3 significantly weakened tumorigenicity. Other studies have shown that CCT3 knockdown leads to microtubule protein deficiency, which is one of the key mechanisms inhibiting cell motility and arresting the cell cycle. CCT3 knockdown also impairs LUAD progression by reducing the synthesis of eukaryotic translation initiation factor 3 (EIF3G) and weakening glycolytic function, leading to reduced ATP production.^[20]^ Additionally, CCT3 promotes LUAD cell proliferation and growth by inhibiting Slc7a11-mediated ferroptosis and positively regulating the AKT signaling pathway.^[31]^ Further research on CCT3 in LUAD revealed that CCT3 mRNA expression is significantly higher in p53-mutant LUAD cohorts compared to the p53 wild-type group. GeneMANIA and String database analyses identified a group of CCT3-associated genes, which are involved in the assembly and stabilization of proteins that participate in cytoskeletal homeostasis, DNA repair, and protein methylation. CCT3 expression was positively correlated with infiltrating Th2 cells, but negatively correlated with mast cells and immature dendritic cells. Gene Set Enrichment Analysis showed that CCT3 is enriched in pathways related to the cell cycle, protein export, proteasome, ribosome, JAK/STAT, B-cell receptor, T-cell receptor, and toll-like receptor signaling pathways.^[78]^ These findings suggest that CCT3 is not only a potential molecular marker and therapeutic target in LUAD, but also a key immunoregulatory factor. However, the underlying mechanisms of CCT3’s involvement in LUAD require further investigation.

### 4.3. CCT3 and breast cancer

Breast cancer is one of the leading causes of cancer-related deaths among women worldwide, responsible for approximately 685,000 deaths in 2020. In developed regions, the 5-year survival rate exceeds 80%, largely due to early detection, diagnosis, and access to effective treatment.^[79]^ Bioinformatics analysis of CCT subunit expression in breast cancer reveals that CCT2, CCT3, CCT5, CCT6A, CCT7, and CCT8 are significantly upregulated, with their expression levels positively correlated with tumor stage. Except for CCT6A, all of these subunits are significantly associated with overall survival.^[80]^ Xu G et al^[50]^ demonstrated that CCT3 influences the proliferation, cell cycle, and metastatic ability of breast cancer cells through the NF-κB-p65 signaling pathway. In the same year, experiments in nude mice showed that tumors with silenced CCT3 were smaller in both weight and volume compared to control groups (non-silenced). Furthermore, CCT3 knockdown significantly reduced Wnt signaling in breast cancer cells and increased miR-223 expression. When CCT3 was upregulated, nuclear β-catenin levels were significantly elevated, suggesting that CCT3 may promote breast cancer progression by directly interacting with miR-223, thereby diminishing miR-223’s regulatory role in the Wnt/β-catenin pathway.^[26]^ Additionally, CCT3 may influence apoptosis in highly invasive breast cancer cells through interactions with cell cycle signaling networks and pyruvate carboxylase.^[81]^ Another study revealed that CCT3 silencing resulted in mitochondrial membrane potential disruption, increased intracellular reactive oxygen species levels, and elevated p53 expression, altering energy metabolism-related factors. This suggests that miRNA-mediated CCT3 knockdown induces apoptosis by disrupting intracellular reactive oxygen species homeostasis, affecting the distribution of free amino acids in energy metabolism, and causing DNA damage.^[27]^ These findings indicate that CCT3-regulated pathways may offer potential therapeutic targets for breast cancer treatment.

### 4.4. CCT3 and cervical cancer

Cervical cancer is the fourth most common cancer among women worldwide, accounting for nearly 8% of all female cancer deaths annually. In 2020, it was responsible for approximately 342,000 deaths globally. Nearly all cases of cervical cancer are caused by HPV infection, with only a small fraction unrelated to HPV.^[82]^ The primary treatment options currently include surgery, radiotherapy, and chemotherapy. However, screening and HPV vaccination efforts are difficult to implement in low- and middle-income countries, and the prognosis for patients with advanced or recurrent cervical cancer remains poor. Therefore, there is an urgent need to explore new molecular markers to improve detection rates and enhance patient outcomes. Dou et al,^[35]^ through Gene Expression Profiling Interactive Analysis database analysis, found that CCT3 expression is upregulated in cervical squamous cell carcinoma and adenocarcinoma (CESC) and is associated with poor prognosis. Silencing CCT3 inhibited the cell cycle and invasiveness of CESC cells while inducing apoptosis. This regulation was associated with multiple pathways, with overexpression of FN1 significantly rescuing the inhibition of CESC cell proliferation caused by CCT3 knockdown, suggesting that CCT3 upregulation promotes cervical cancer progression through FN1. Another study showed that CCT3 expression is significantly elevated in cervical cancer tissues compared to adjacent normal tissues and is associated with HPV-16/18 infection, tumor grade, and positive lymph node status. High CCT3 expression correlates with poor prognosis and demonstrates strong predictive potential.^[83]^ These findings suggest that CCT3 may serve as a potential biomarker and a promising therapeutic target for cervical cancer.

### 4.5. CCT3 and MM

MM is a malignancy of plasma cells, with an increasing incidence over time. Risk factors for MM include age, race, gender, and family history. The disease is characterized by monoclonal proliferation of plasma cells, leading to the production of monoclonal antibodies and end-organ damage, which causes hypercalcemia, kidney impairment, anemia, and bone lesions.^[84]^ CCT3 is highly expressed in MM tissues and is associated with poor prognosis. Its expression increases progressively with the malignancy of MM, and CCT3 may promote tumor progression by regulating Myelocytomatosis oncogene through the JAK–STAT3 signaling pathway.^[23]^ Liu D et al^[85]^ demonstrated that circ-CCT3 is upregulated in bortezomib-resistant MM cells. Silencing circ-CCT3 enhanced the sensitivity of bortezomib-resistant MM cells to bortezomib. Circ-CCT3 mediates bortezomib resistance by modulating the miR-223-3p/BRD4 pathway, providing a novel potential target for overcoming chemoresistance in MM. Thus, CCT3 may serve as a potential therapeutic target in MM.

### 4.6. CCT3 and other tumors

Currently, extensive research has been conducted on CCT3 in HCC, lung cancer, breast cancer, cervical cancer, and MM. However, CCT3 has also been studied in other cancers. CCT3 is highly expressed in many malignant tumors and is associated with tumor pathological stage, grade, survival, prognosis, and the immune microenvironment.^[86]^ Li LJ et al^[29]^ found that CCT3 is highly expressed in gastric cancer tissues, primarily in the cytoplasm. Silencing CCT3 inhibits tumor cell growth, proliferation, and promotes apoptosis. In nude mouse experiments, knocking down CCT3 suppressed tumor growth, significantly reducing tumor volume and weight. In thyroid cancer, both CCT3 mRNA and protein levels are highly expressed, and silencing CCT3 reduces cell viability, proliferation, and cell cycle progression, while inducing apoptosis.^[87]^ In colorectal cancer (CRC), circCCT3 is highly expressed and associated with advanced-stage CRC. Patients with low expression of circCCT3 have a higher survival rate. miR-613 is the target of circCCT3, responsible for regulating CRC cell invasion and apoptosis. CircCCT3 promotes tumor metastasis by regulating VEGFA and the Wnt signaling pathway through miR-613.^[55]^ In head and neck squamous cell carcinoma, CCT3 mRNA and protein are upregulated and correlate with clinical characteristics and survival. Knocking down CCT3 inhibits cell growth, invasion, and induces cell death.^[63]^ In melanoma, high expression of CCT3 is related to clinical staging, and in nude mouse experiments, silencing CCT3 significantly inhibits tumor growth. CCT3 knockdown suppresses cell proliferation and cell cycle progression (G0/G1 phase arrest, with a reduced percentage of cells in the S or G2/M phase) and induces apoptosis, while significantly decreasing CDK1 expression. This indicates that CCT3 depletion may block melanoma progression by downregulating CDK1.^[34]^ Luo L et al^[88]^ demonstrated that circCCT3 is highly expressed in bladder cancer, with diagnostic sensitivity of 79.5% and specificity of 65.6% in plasma tests, suggesting that plasma circCCT3 has potential diagnostic value for bladder cancer. CircCCT3 expression correlates with survival, pathological stage, and tumor grade. Silencing circCCT3 inhibits cell growth, proliferation, and induces apoptosis while increasing miR-135a-5p expression. CircCCT3 influences bladder cancer progression by regulating the miR-135a-5p/PP2A axis.

## 5. Conclusion

CCT has the most complex structure among molecular chaperones and plays a crucial role in assisting proper protein folding, as well as refolding or degrading misfolded proteins. It is involved in various cellular processes such as DNA replication, transcription, intracellular signal transduction, and cytoskeletal function. When CCT expression becomes dysregulated, it can lead to loss of protein structure and function, resulting in uncontrolled rapid cell growth and proliferation, which can eventually drive tumorigenesis. For CCT to perform its functions, it requires the formation of an oligomeric complex consisting of 8 subunits (CCT1–8). Each of these subunits is essential and critical for CCT’s role in promoting tumorigenesis. The loss or overexpression of a single CCT subunit can impact the biological function of the entire oligomeric complex. While CCT subunits are important for tumor development, further investigation is needed to explore whether CCT interacts with specific oncogenic factors through the entire complex or via individual subunits.

CCT3, as one of the subunits of the CCT, plays a key role in tumor cell growth, proliferation, cell cycle regulation, and survival. It is highly expressed in various malignancies and is closely associated with cellular phenotypes, clinical characteristics, pathological stages, and prognosis. CCT3 promotes tumor progression through multiple signaling pathways and is linked to immune cell infiltration and immune checkpoint expression in the tumor microenvironment, presenting a new potential target for overcoming drug resistance in various cancers. CCT3 shows promise as a diagnostic biomarker and may be a novel target for immunotherapy across multiple tumor types. In this article, we introduce the structure and function of CCT and CCT3, discussing the correlation between CCT3 and oncogenic factors, as well as the research progress of CCT3 in various cancers. This review provides crucial insights for identifying new biomarkers and therapeutic targets in cancer treatment.

## Author contributions

**Conceptualization:** Yun-Feng Bai, Xiao-Hui Shi, Mo-Lemei Zhang.

**Data curation:** Yun-Feng Bai, Mo-Lemei Zhang.

**Formal analysis:** Yun-Feng Bai, Jiahui Gu, Ta-La Bai, Yin-Bao Bai.

**Funding acquisition:** Xiao-Hui Shi.

**Investigation:** Yun-Feng Bai, Jiahui Gu, Ta-La Bai, Yin-Bao Bai.

**Resources:** Jiahui Gu.

**Supervision:** Xiao-Hui Shi, Ta-La Bai, Yin-Bao Bai.

**Writing – original draft:** Yun-Feng Bai, Mo-Lemei Zhang.

**Writing – review & editing:** Xiao-Hui Shi.
